# Medical Society of the County of Albany, Semi-Monthly Meetings, Dec. 17, 1872 and Jan. 8, 1873

**Published:** 1873-01

**Authors:** F. C. Curtis

**Affiliations:** Secretary


ART. III.—*Medical Society of the County of Albany.—Semi-Monthly Meetings, December* 17, 1872, *and January* 8, 1873.

Reported by F. C. Curtis, M. D., Secretary.

The Society met at the usual place and hour, in the city building, and was called to order by the President, Dr. Van Derveer. There were present about thirty members.

After the minutes had been read and approved, the death of Dr. J. F. Crouse, a late member of the Society, was referred to, and a committee appointed to draw up suitable resolutions in regard to it, consisting of Drs. J. S. Bailey, Lewis and Bigelow.

A paper was then read on the subject of Hydrorrhoea, by Dr. Quackenbush.

The term he defined as one applied to those discharges of water occurring in the later months of pregnancy, which are neither preceded nor followed by uterine contractions, and do not interfere with the progress of the gestation to full term.

The character of this fluid is similar to, if not identical with, the amniotic fluid. It may make its appearance, without premonition, by a sudden gush, in considerable quantity, or it may dribble away drop by drop. In either event it is not attended with uterine pains or other symptoms of approaching abortion.

The views which various authors have entertained as to the nature and source of these *false waters,* as they are sometimes called, have been numerous and diverse. Bœhmer supposed they were contained between the chorion and amnion, and were discharged by a rupture of the chorion; Mauriceau considered them to be amniotic fluid, draining away from the interior of the amnion by a rupture of the membranes high up above the neck; others have explained it as an œdema of the uterus itself. Carl Braun, of Vienna, has made two kinds of fluid accumulation; one between the uterine walls and chorion, called *hydrometra ascitica*, the other between the chorion and amnion. The report of Prof. Doughty, of Augusta Medical College, in the *American Journal of Obstetrics*, was also referred to, of a case in which a watery discharge took place in the fifth month, continuing until the seventh month, when she give birth to twins, the sac of one fœtus being almost empty of fluid and no signs of a rupture of the membranes having taken place at any point. To the view of the author that this was an ordinary case of hydrorrhœa, exception was taken.

Professor QUACKENBUSH then proceeded to state that eight years ago, while exhibiting a placenta to the medical class, he discovered on its fœtal surface a cyst containing about two ounces of fluid similar in character to the amniotic. He has since noted others, occurring always on the fœtal surface. They are not cystic tumors proper, but a simple accumulation of fluid between the membranes from arrest of transudation. The source of all fluids and nourishments to the fœtus is the placenta. This is the objection to the *hydrometra ascitica* of Carl Braun, which view is untenable because of the lack of a surface and tissue to secrete the fluid. The placenta is a double organ, having two distinct parts—the fœtal, which is vascular, and the maternal, which is nutritive and secretory. The false waters as well as the true can only originate through the placenta. Or even should it form between the uterine walls and the chorion, it would necessarily dribble away drop by drop, because the membrane is too slightly adherent to hold it in any quantity.

His own view he expressed as follows: The fluid, failing to transude through the membranes, accumulates in the form of a cyst on the fœtal surface of the placenta; as it increases in quantity the cyst distends until it reaches the periphery of the placenta, when rupture of its walls takes place from lack of support, and the fluid escapes between the chorion and the walls of the uterus.

He presented the following case of Hydrorrhœa: Mrs. B., in the seventh month of her fifth pregnancy, while walking in the street, felt a sudden gush of water, which she supposed was due to a rupture of the membranes. On stepping aside she noticed the pavement quite wet with the flow that had occurred. She hastened home, and was seen soon after by Dr. Quackenbush. He discovered no symptoms of approaching labor, and did not find anything other than it should be. Rest was enjoined, and she went on to full term, and was delivered naturally, the bag of waters forming as usual.

At the conclusion of the paper a perfect specimen of a placenta and membranes was presented, on which was shown the lax connection between the chorion and amnion, and by it the view taken was explained.

On the motion of Dr. W. H. BAILEY, it was voted that the paper be transmitted to the State Society as a communication from the County Society.

Dr. FOWLER reported a case in which a patient, in the sixth month of pregnancy, fell backward from a chair with force. At the time much fluid escaped from the uterus, and when seen the patient was still lying on the floor where she had fallen. The os was not dilated, and she was left after giving an opiate. From the time of her fall she was relieved of a dyspnœa which had troubled her before it. She was delivered naturally at full term. The supposition was that, in this case, there was a rupture of the membranes.

Dr. SWINBURNE raised the question whether the liquor amnii can accumulate again after being discharged; he believed that it could.

Dr. QUACKENBUSH differed from this view.

Others took part in the discussion of the paper.

After a short recess, Dr. DEVOL spoke upon the subject of "Favorite Remedies," giving a good number of valuable combinations of medicines, which recommended themselves to the thoughtful consideration of the Society. He mentioned those only which in a long practice he had found especially useful, detailing the circumstances and conditions in which he employed them.

Dr. BIGELOW said that he was familiar with many of Dr. Devol's formulæ, and they had been very useful to him. The subject of stimulants had been alluded to; it was one growing in interest and prominence before the public.

On the motion of Dr. SWINBURNE, it was voted that Dr. Devol be requested to present a paper on the subject of stimulants at the next meeting.

On motion, the Society adjourned.

---

SEMI-MONTHLY MEETING, January 8, 1873.

The Society met at the city building at 8 o'clock P. M., and was called to order by the President, Dr. Van Derveer. There were twenty-two members present, besides other medical gentlemen.

Dr. QUACKENBUSH, after remarking upon the approaching meeting of the State Medical Society here in February, made a motion that an entertainment be given to the members of it by our County Society, and a committee of six be appointed to attend to the business of providing it.

The motion was carried without debate.

The committee appointed consists of Drs. Van Derveer (*ex-officio*), Quackenbush, Boyd, Thomas Hun, Swinburne, and W. H. Bailey.

Dr. DEVOL read a paper entitled "Intoxicants Dispensed with in Medical Practice."

Alcohol is the genus of which all intoxicants are species. Absolute alcohol is composed of three simples, thus: Carbon 4 equivalents, hydrogen 6, and oxygen 2; or, by volume, carbon 4, hydrogen 6, and oxygen 1. Its specific gravity is 80-100. It boils at 172° F. It is not congealed at 166° below. It is the product of death and decomposition. Alcohol is the union of three elements in such relations and proportions as were never found in earth, or air, or water. Alcohol is absolutely destitute of nourishment, and never can be assimilated in the animal constitution. It is a foreign body, and is irritant in the animal body. The extent to which spirituous liquors are used in the sickroom, and the results of their use, only need to be known to create a correct public sentiment on this subject, to suppress their use, and withhold patronage from rum doctors. Employment of intoxicants in the treatment of the sick may be considered from different standpoints. It has an historic aspect, a scientific aspect, a religious aspect, a social aspect, a temperance aspect, and a medical aspect. There is no occasion for the employment of spirituous liquors in the treatment of the sick. There is ample provision in our materia medica for all supposable cases where stimulants are indicated, exclusive of the whole list of intoxicants. In palliation of the use of spirituous liquors in the sick-room, St. Paul is quoted, recommending a little wine for the stomach's sake. Suffice it to say, what everybody ought to know, that the wine he recommended was the unfermented juice of the grape, called in the Scriptures the "best wine."

According to the latest and best authorities in medical literature and science, it is demonstrated that alcoholics are only hurtful in medical practice. I know full well that this sentiment, and whoever asserts it, will be very unpopular in Albany. I presume, nevertheless, to say I discard the whole brood of intoxicants—rum, gin, brandy, bourbon, and beer—and employ non-intoxicant stimulants as substitutes. I dare not prescribe brandy and whiskey and make my patients drunkards, as thousands are so destroyed in Albany and everywhere. Substitutes for intoxicating liquors are derived in great numbers and variety from officinal stimulants, carminatives, and antispasmodics, and completely supplant them. It has been asserted that the innate normal constitution demands, and should have, intoxicants; whereas the appetite for them is adventitious, artificial, habitual, and unnatural. Whoever would be a drunkard must learn to love what he at first loathes and abhors. Mankind monopolizes drunkenness; angels above us and the hogs beneath us decline it in disgust. Alcoholism, or drunkenness, is anesthesia; that is, the subject is strengthless and insensible; all the wheels of life but one or two stand still. Inebriation is a disease—a species of insanity. A drunkard is demoralized generally. He lies in a stupor, with stertorous breathing; he may recover or he may not. The engorged brain may give way, and epilepsy or apoplexy close the scene. But for the division of the nervous system into sentient, motor, and sympathetic nerves, the first drunken debauch would be fatal. The indulgence is at a fearful hazard! Any person assumes a fearful responsibility who uses intoxicants as a beverage or a medicine. A double and terrible criminality attaches itself to the doctor or grog-shop-keeper who holds the cup to his neighbor's lip. Human depravity doubly depraved. Physicians have an undisputed right to be as wise and good as other men.

The fact is patent as it is deplorable that physicians, by prescribing spirituous liquors for the sick, become responsible for a vast amount of drunkenness! Not a few in the medical profession have, in the main, given up the practice of medicine, stopped reading, and gone to prescribing bourbon and brandy to their credulous and much injured patients. Women, especially, are led into drunkenness in this way. For this reason there are, as I believe, more drunken women than men. They are induced to drink while nursing, and hence the infant drinks alcohol in the milk. There is an utter recklessness about taking quack medicines such as Plantation Bitters, Vinegar Bitters, Townsend's Sarsaparilla, Winslow's Soothing Syrup, Female Pills, and every conceivable description of catch-penny empirical frauds. Such audacity and trifling with nostrums and life and health is most absurd and deplorable. If a desperate fanatic choose this mode of suicide he must abide the consequences. Doctors beware! I do not, however, object to tinctures when the doses contain so little alcohol as to make no perceptible impression upon the patient. The drug so prepared is the medicine; the alcohol is merely the vehicle. Rum doctors destroy thousands whom their fellow-trades of the dram-shops never could reach. Innocent children and members of Christian churches and members of the gospel are being drugged and dosed with bourbon and brandy who would not go into a rumsellers den or touch their bane except it were prescribed by their doctors. Not unfrequently staunch temperance men and devoted Christians die of *delirium tremens,* for which they are not in the least responsible. Rum doctors have the undivided, unenviable responsibility of these homicides. The rum doctor's prescription for consumption is cod-liver oil, bourbon whiskey, and a journey to the country. The patient must be taking something. Whisky will keep up the delusion that he is getting better. He is told that his complaint is curable; it is only a slight cold; the blood he coughs up is from the head or throat; the matter he expectorates is only a little mucous; his night-sweats are owing to a change of the weather. Finally he is sent away on a journey. When the patient's eyes are closed his friends eyes are opened; the trickery is exposed; the curtain is dropped; all confidence in the doctor is lost; he is discarded forever; served him right.

Alcoholics do not aid digestion; on the contrary, they impair digestion by precipitating the pepsin, the grastric juice, the digesting fluid. In beef tea and brandy beef tea is the remedy; in hot brandy sling, hot water is the remedy. Cumulative poisons produce mercurialism, cinchonism, egotism, iodism, and alcoholism, which is anesthesia or drunkenness. In extensive hospital practice in Europe and this country *delirium tremens*, treated with alcoholics, 50 per cent. die; treated with warm baths and nutritious diet, without opium or alcohol, one in a hundred die. Alcoholics dispensed with in the treatment of fevers in Naples, mortality was reduced from 28 to 7 per cent.; the same results in fever hospitals in Glasgow; in British and French hospitals the same results. Ten thousand drunkards died of cholera in England and Ireland, and scarcely ever a temperate man. Cholera kills drunkards!

Substitution of tea and coffee for rum in the army and navy is a good thing for sailors and soldiers. Some people wonder at the frequency of apoplexy, induration of the spleen and liver and fatty degeneration of the heart; also the prevalence of idiocy and insanity, of scrofula and consumption. No marvel; intemperance explains the whole.

No one who is capable of feeling the responsibility of a parent or physician would ever drug children with the nostrums in the shops as *Drops, Elixirs, Cordials, Balsams* of life, Winslow's Soothing Syrup. Beer-guzzling and whisky-drinking mothers give their babes alcohol enough through the lacteal secretion of the mammary gland to make them habitual drunkards before they can speak or walk, and the nurse keeps concealed about her the drops that "make the baby sleep." Drunkenness prevails in the nursery! The paroxyisms of infantile drunkenness are well defined. The demand for the *drops* is urged with petulent cries that nothing else can suppress.

Such is the effect of narcotics on the infantile generation that parental authority is ignored; age no longer commands respect; civil authority is set at defiance, and all restraint,—parental, social, religious or civil—is desperately resisted. Our future is ominous enough. We see in the not far distant future more and more ruffianism, and ignorance, and vice, and penitentiaries, and cemeteries full. Dirks, bowie-knives, slung-shots and revolvers will be more and more fashionable, and suicides, robberies and murders will, indefinitely, be multiplied; the majesty of the law and the ends of justice lost in the corruption and bribery sanctioned by position or gold.

Dr. BECKETT asked what authority there is that alcohol is not a nutrient and does not promote assimilation. He would differ from some of the statements in the paper. It is hardly possible to find anything to take the place of alcohol in medicine. In his own recent sickness with pneumonia, he took a pint a day of Bourbon whisky. He took other stimulants, as carbonate of ammonia, quinine, &c., but these often added to his sufferings, which consisted of high fever and intense headache, while the whisky always diminished them. The burning pain in his head was often such that it was necessary to pour ice water over it, but whisky would, after a time, relieve it and put him to sleep. Does its use in such cases increase intemperance? He thought it impossible to make men drunkards by using it in acute cases. In his own case the taste was unpleasant while taking it, and since recovery he felt no desire for it. Opium might with equal propriety be rejected from the Pharmacopœia. The fact that alcohol has a chemical origin and does not exist in nature, is no stronger an argument against it than against many other drugs. As to its increasing the temperature necessarily, a drunken man will freeze sooner than one sober.

Dr. SWINBURNE said that most of the poorer classes in Great Britain drink gin and beer, which would account for the large proportion there of deaths from cholera among habitual drinkers, compared with those who totally abstained from its use. He thought the latter class as likely to take the disease as the former. Liquors in England cost more than all the necessaries of life. On the continent of Europe, where the use of light wines is universal, he seldom saw in public gatherings, in parks and places of amusement, a person intoxicated. If we are to be a temperate people, it must be by the same general use of a mild beverage. Its medical use requires discretion; there are those who can and those who cannot bear alcohol in fevers.

Dr. C. S. HOYT referred to a paper on cholera by Dr. Coventry, written in 1853, and said that cholera is no more severe among the intemperate, who continued to drink, than among the temperate, but was worse with those who attempted to reform. In the army it was considered indispensable. In lunatic asylums it is used extensively as a sedative in certain forms of insanity. In nervous affections, accompanied with wasting of tissues, it is valuble, especially combined with coffee, acting here to retard disintegration of tissue. His experience of its use in fever confirmed Dr. Beckett's. He agreed with Dr. Swinburne, that after its use in acute diseases, there was a disgust felt for it. He knew many cases where it had been given in the army hospitals in very large quantities, and he knew of none of these who continued its use now. Those who use quack preparations, such as "Plantation Bitters," are very apt by this means to become intemperate.

Dr. BLATNER was asked to give the result of his observation in Germany of the use of alcoholics. He said that while at Leipsic, cholera was threatened, and red wine was recommended to be used freely by Wunderlich and other professors. It was followed by good effects, attributed to its nutrient and astringent qualities. He saw light wine and beer used universally by German students in Berlin, Vienna, and other university towns, as well as by the professors, and all are familiar with the amount of mental work they accomplish. He thought the lower classes could hardly do without beer, as in it they found an article of food at once cheap and nutritious. There are many who live entirely on black bread and beer.

Dr. LORENZO HALE reported a case of Syphilitic Paraplegia, and presented the pathological specimen of the case, showing in a section of the spinal cord taken from the lumbar region a gumma about three-eighths of an inch in diameter, located opposite the third lumbar vertebra. The patient, who was 31 years of age at the time of death, had the primary sore five years ago. He was first seen by Dr. Hale two years ago, then having sarcocele, both testicles being affected. He presented other symptoms of constitutional syphilis. Under Iodide of potassium he soon improved so as to resume his occupation.

A year ago paralysis of the right leg came on gradually and became total. Sensation and motion were afterward impaired in the left leg. The right leg could be pinched without pain, but a severe jar caused pain at the hip and knee. Three weeks before death he became very feeble and passed into a semi-unconscious condition, the pupils being somewhat contracted. There was incontinence of urine and fœces, ascites and pleuritic effusion. The paraplegia was complete of both sides. Toward the last his pulse rose from 80 up to 120, respiration was accelerated and temperature high.

"According to Ricord, gummy tumors never appear earlier than six months after contagion, and usually several years. The microscopical appearance of them are thus described by Lebert:

"A thin section of the tumor is found to consist of loose fibrous tissue, made up of pale elastic fibres, inclosing in their interspaces a homogeneous granular substance, the elements of which are less adherent to each other than in deposits of true tubercle. The granulated cells or corpuscles do not exceed in size m,005. They are rounded and contain an irregularly granulated substance. Some of the larger cells have pale and irregular walls, and appear to contain a rounded neucleus."

Robin says that "these cells are made up of rounded nuclei, belonging to fibro-plastic cells, or *cytoblastions*; of a finely granular, semi-transparent and amorphous substance, and finally of isolated fibres and a few capillary blood vessels. They may occur on all the external parts of the body, (spinal cord not mentioned). I have found no case recorded in which a gumma has been found in the spinal cord, post mortem, though there are cases in which it has been diagnosticated."

Hammond says "intra-spinal tumors may result from the syphilitic, scrofulous and cancerous diathesis." He relates a case of paraplegia of supposed syphilitic origin which was cured by iodide of potassium.

Durkee says "they have been found in the substance of the skin, in the subcutaneous cellular tissue, muscles, tendons, epididymis, testicle, liver, lungs, brain, bones, etc.".

Dr. Van Derveer presented a specimen of mono bromide of camphor. This, he said, has been spoken of for the past ten years in Germany, and thought highly of. His own experience with it is favorable, though not large. It is adapted to delirium tremens, hysteria and convulsions of children, and often operates with marked success. The dose is from one to four grains. It is not expensive.

Dr. Morgan reported a case of cancer of the uterus, and presented the pathological specimen. The patient was aged 56 at the time of death. A remarkable fact was that but little pain had attended its development, there being times, however, when it was severe. She had slept well and appetite good, and her strength continued remarkably until a week before death, so that she assisted in the household duties of the Home for the Friendless, where she was. For the past nine months there has been a constant discharge of a most offensive odor. Post mortem, it was found that the os, neck and half the body of the uterus was affected, especially posteriorily and on the left side, being much broken down; the walls of the vagina and the rectum were affected with more recent deposits. There was no perforation into the pelvic cavity.

Dr. Lansing presented a specimen illustrating pericarditis. The heart was enlarged and the pericardium everywhere adherent. The case shows the difficulty of diagnosticating pericarditis often. Its existence here was not diagnosticated; there was no murmur; the heart sounds were indistinct and irregular, which with the enlarged area of dulness lead to the opinion that it was pericardial effusion.

Last September, three months ago, the patient was exposed to cold, and there followed swelling of the extremities and other symptoms of Bright's disease. The urine was albuminous. There was afterwards dyspnoea and cough without expectoration. There was no history of rheumatism.

The post mortem showed, besides this condition of the heart, the kidneys in the second stage of Bright's disease.

The case raises the question whether in such cases the serous inflammation is a primary affection or secondary to the disease of the kidney. He thought the latter view was the correct one. Idiopathic peritonitis is very rare.

